# *Drosophila* larval motor patterning relies on regulated alternative splicing of *Dscam2*

**DOI:** 10.3389/fnmol.2024.1415207

**Published:** 2024-07-18

**Authors:** G. Lorenzo Odierna, Sarah K. Kerwin, Grace Ji-eun Shin, S. Sean Millard

**Affiliations:** ^1^School of Biomedical Sciences, The University of Queensland, Brisbane, QLD, Australia; ^2^Department of Neurology, The Ohio State University Wexner Medical Center, Columbus, OH, United States; ^3^The Neuroscience Research Institute, The Ohio State University, Columbus, OH, United States; ^4^The Ohio State University Comprehensive Cancer Center, Columbus, OH, United States

**Keywords:** *Dscam2*, *Drosophila*, alternative splicing, fictive locomotion, motor neuron, dendrite targeting, looper, A02

## Abstract

Recent studies capitalizing on the newly complete nanometer-resolution *Drosophila* larval connectome have made significant advances in identifying the structural basis of motor patterning. However, the molecular mechanisms utilized by neurons to wire these circuits remain poorly understood. In this study we explore how cell-specific expression of two *Dscam2* isoforms, which mediate isoform-specific homophilic binding, contributes to motor patterning and output of *Drosophila* larvae. Ablating *Dscam2* isoform diversity resulted in impaired locomotion. Electrophysiological assessment at the neuromuscular junction during fictive locomotion indicated that this behavioral defect was largely caused by weaker bouts of motor neuron activity. Morphological analyses of single motor neurons using MultiColour FlpOut revealed severe errors in dendrite arborization and assessment of cholinergic and GABAergic projections to the motor domain revealed altered morphology of interneuron processes. Loss of *Dscam2* did not affect locomotor output, motor neuron activation or dendrite targeting. Our findings thus suggest that locomotor circuit phenotypes arise specifically from inappropriate Dscam2 interactions between premotor interneurons and motor neurons when they express the same isoform. Indeed, we report here that first-order premotor interneurons express *Dscam2A*. Since motor neurons express *Dscam2B*, our results provide evidence that *Dscam2* isoform expression alternates between synaptic partners in the nerve cord. Our study demonstrates the importance of cell-specific alternative splicing in establishing the circuitry that underlies neuromotor patterning without inducing unwanted intercellular interactions.

## 1 Introduction

Neuromotor patterning underlies many key aspects of animal behavior including locomotion. Understanding the cell and molecular basis of neuromotor patterning is therefore a key goal in deciphering nervous system function. Fruit flies (*Drosophila melanogaster*) provide a unique platform to understand how neuromotor patterning arises due to their genetic tractability, relatively simple nervous system, and stereotypic behavioral output. The recent completion of a full, nanometer-resolution (3.8 × 3.8 × 50 nm) map of the *Drosophila* larval brain and ventral nerve cord (VNC) has provided significant progress toward unveiling the structural basis for motor patterning (Winding et al., 2023). Despite this advancement, much remains to be understood. In fact, the rules and molecular toolkits used by VNC neurons to generate their complex circuits remain largely unexplored.

In this study, we examined the role of alternative splicing in the patterned network output of *Drosophila* larval locomotor circuits. We did so by investigating the *Down Syndrome Cell Adhesion Molecule 2* (*Dscam2*) gene, which undergoes alternative splicing to wire the developing adult fly visual system (Lah et al., 2014). Dscam2 proteins are transmembrane cell recognition molecules that engage in short-range homophilic interactions to induce repulsion (Millard et al., 2007, 2010) or adhesion (Tadros et al., 2016) between neurons. The *Dscam2* gene is alternatively spliced to produce two biochemically distinct extracellular isoforms, known as Dscam2A and Dscam2B, which mediate isoform-specific homophilic binding similar to the Dscam2 paralogue Dscam1 (Wojtowicz et al., 2004; Millard et al., 2007). *Dscam2* alternative splicing is regulated at a cell-specific level; neurons typically express either *Dscam2A* or *Dscam2B* exclusively (Lah et al., 2014; Li and Sean Millard, 2019). We recently discovered that the larval VNC hosts cell bodies and projections of many *Dscam2A-* and *Dscam2B*-positive neurons (Odierna et al., 2020). *Dscam2B* is specifically expressed in a subset of peripheral (motor and sensory) neurons, whereas both *Dscam2A* and *Dscam2B* are expressed in the VNC. Although *Dscam2B* is expressed in small populations of neurons as early as embryonic stage 16 (1-2 neurons per VNC segment), the expression of both isoforms increases dramatically in many VNC neurons before hatching (Odierna et al., 2020). Because it switches on so late, *Dscam2* is unlikely to be involved in early developmental processes such as axon outgrowth or dendritogenesis, as is the case for similar cell surface molecules (Sánchez-Soriano and Prokop, 2005; Kamiyama et al., 2015). Instead, the proximity of the expression peak to hatching implicates *Dscam2* in refining the circuitry responsible for the functional execution of behavioral programs in larvae (Crisp et al., 2008). As such, *Dscam2* serves as a good candidate to study how alternative splicing of a key wiring molecule contributes to motor patterning in *Drosophila* larvae.

## 2 Methods

### 2.1 Fly stocks and genetics

Flies were reared at room temperature and humidity on standard cornmeal medium. All lines used in this study were on a *w^1118^* background and are outlined in Supplementary Table S1. Third instar larvae of either sex were selected for all experiments.

### 2.2 Cloning of Dscam2-V5 BAC

The V5-tagged *Dscam2* BAC clone was created by modifying CH321-40122,^1^ which contains 80 kb of *Dscam2* genomic sequence and an attB site for site-specific integration. Exon 5 of *Dscam2* within this BAC was modified using bacterial recombineering. The first step was replacing the exon 5 region with Kana-RpsL to allow for both positive and negative selection (Wang et al., 2009). This was done by amplifying Kana-RpsL (pSK-KanaRpsL, Addgene) using primers SM290/SM291 which flanked the cassette with BamHI sites. A *Dscam2* replacement cassette was synthesized (DNA 2.0) containing a 55 bp homologous left arm (intronic sequence)-BamHI, a 33 bp B3 recombinase site, 87 bp of the intron preceding exon 5, 160 bp of exon 5 including a V5 tag, 31 bp of intronic sequence following exon 5, a 33 bp B3 recombinase site followed by BamHI, and a 51 bp homologous right arm consisting of intronic sequence. The V5 tag was placed within a hydrophilic region of exon 5 that is predicted to be exposed to solvent (SHRGIISPSVVEHTV5AHVQV). The Dscam2 replacement cassette was used to replace the Kana-RpsL cassette using BamHI sites. The Kana-RpsL cassette was integrated into the CH321-40122 BAC after transforming SW102 cells using recombineering procedures and selecting on kanamycin (25μg/mL). The Kana-RpsL insert was replaced with the Dscam2 V5 sequence using a similar procedure and selection on streptomycin (1 mg/mL). The modified region of the BAC was sequenced and then integrated into the VK37 attP site on chromosome 2 (Genetivision).

SM290—GCTGTCGGATCCAGCTTCACGCTGCCGCAAGCACTCAG.
SM291—CATTCAGGATCCGGGGTGGGCGAAGAACTCCAGCATGA.

### 2.3 CRISPR gene editing

*Dscam2^GFP-FLAG^* insertion was generated using one gRNA and a template through CRISPR HDR. The GFP-FLAG sequence was derived from the Bellen Lab (EGFP-FlAsH-StrepII-TEV-3xFlag; Nagarkar-Jaiswal et al., 2015). The template was synthesized and inserted into cloning vector pUC57 (General Biosystems). Template and gRNA were co-injected into embryos (Actin-Cas9, Lig4) and confirmed through sanger sequencing.

gRNA *Dscam2^exon20^*—5′ GTCGAGTCCTCCAACCAGCACGA 3′.

### 2.4 Locomotion grid assay

Larval locomotion was assayed according to methods described by Nichols et al. (2012). Larvae were transferred from their vials onto a 35 mm plate containing nutrient-devoid agar (1% w/v in deionized water) over graph paper. After 1–2 min acclimation, the larvae were filmed under free roaming conditions for 60–90 s using a Nikon Digital sight Ds-Ri1 camera mounted onto an Olympus SZX12 microscope. Analysis was performed by counting the number of grid lines passed by the larvae within 60 s as well as the duration spent within each individual grid. We also measured the number of directional changes taken by larvae during locomotion. This was defined as any time larvae performed a head swing during locomotion and continued moving down the new path set by the head swing. We also measured head swings performed while the larvae were immobile. For all behavioral experiments, both recording and analysis were blinded.

### 2.5 Fictive locomotion assay and synaptic bursting analysis

Larvae were filleted and pinned open on sylgard dishes in HL3 (Stewart et al., 1994) containing 1.5 mM Ca^2+^. The central nervous system, including the ventral nerve cord, was left intact. Peripheral nerve fibers, except those projecting to segments A3 and A4, were severed to reduce mechanical disturbances caused by peristaltic contractions. Because most nerves are severed in this preparation, the testing of intersegmental coordination is not possible. The prepared dissection was visualized on a Nikon Eclipse FN1 (Nikon instruments Inc., NY, United States). Muscle 6 in A3 or A4 was impaled using a high resistance (80–100 MΩ) sharp borosilicate electrode filled with 3 M KCH3COO and 3 M KCl (2:1 ratio). Preparations were superfused with HL3 heated to 30°C to elicit robust motor patterns (Kurdyak et al., 1994). Recordings of muscles during spontaneous motor activity were amplified using an Axoclamp 2B amplifier (Molecular Devices) and collected using Labchart 7.0 (AD instruments) at a sampling rate of 2 kHz. Analysis of data was performed in LabChart 7.0 using semi-automated methods. Excitatory junctional potentials (EJPs) were identified using the “Cyclic Measurement” function, marking EJPs above baseline noise/mEJPs via a manually set threshold. Previous studies have used different approaches to define bursting “events,” including qualitative assessment/manual classification or inter-EJP interval (Barclay et al., 2002; Suster et al., 2004; McKiernan, 2013; Karunanithi et al., 2020). In our recordings, we noted only two kinds of activity: (1) Spontaneous or extremely low frequency EJPs and (2) high frequency bursts of EJPs. To separate the spontaneous activity from the bursts, we defined bursting events as consecutive EJPs occurring within 0.3 s of each other. Although the inter-burst interval of EJPs within high frequency bursts is much lower than 0.3 s, we wanted our threshold to be as broad as possible to capture any changes in *Dscam2* mutant larvae. Because the inter-EJP of 0.3 s was relatively long, we occasionally captured longer strings of very low frequency spontaneous EJPs. There were also some instances where high frequency events would occur consisting of very few EJPs. To avoid classifying these as bursts, events that contained fewer than 20 EJPs were excluded from the analysis. Burst “runs” were defined as repeated consecutive bursts within 10 s of each other and only considered if they had 3 or more bursts in a row. In total, the total number of runs and bursts measured per genotype was 39 and 229 for controls, 35 and 228 for *Dscam2^null^*, 25 and 229 for *Dscam2A*, and 11 and 150 for *Dscam2B*.

### 2.6 Immunohistochemistry protocols

Larvae were dissected in calcium-free HL3 following the magnetic body-wall muscle procedure described in Ramachandran and Budnik (2010). Following dissection and fixation in formaldehyde solution (4% wt/vol paraformaldehyde in phosphate buffered saline) for 20 min, immunohistochemistry was performed on larval fillets or ventral nerve cords. Fixed tissue was blocked in phosphate buffered saline (PBS) with 10% goat serum and 0.1% Triton-X and then incubated with primary antibodies overnight. Fillets were then washed in PBS and incubated with secondary antibodies overnight. After thorough washing, fillets were mounted onto glass slides in 70% glycerol and imaged. Antibodies used in this study are outlined in Supplementary Table S2.

### 2.7 Fluorescence image acquisition

Microscopy was performed at the School of Biomedical Sciences and Queensland Brain Institute’s Advanced Microscopy Facility. Images were collected using an Olympus FV1000 upright scanning confocal microscope using 40× air, NA 1.35 60× oil or NA 1.4 100× oil immersion objectives or a spinning-disk confocal system (Marianas; 3I, Inc.) consisting of a Axio Observer Z1 (Carl Zeiss) equipped with a CSU-W1 spinning-disk head (Yokogawa Corporation of America), ORCA-Flash4.0 v2 sCMOS camera (Hamamatsu Photonics), NA 1.4 63× P-Apo objectives. Image acquisition was performed using SlideBook 6.0 (3I, Inc). ChAT/VGAT VNCs were scanned at 2,048 × 2,048 pixels.

### 2.8 Analysis of cholinergic and GABAergic inputs

Triple labeled nerve cords with OK6-GAL4 > GFP, anti-ChAT and anti-dVGAT fluorescence were subjected to automated analysis using ImageJ. We generated a method to create custom masks for each nerve cord based on a combination of OK6-GAL4 > GFP fluorescence and anti-ChAT immunoreactivity (outlined in Supplementary Figure S1). Anti-ChAT immunoreactivity was subjected to Gaussian blurring (sigma = 5) and autothresholded using the “Li” method, and OK6-GAL4-based fluorescence was subjected to gaussian blurring (sigma = 5) and autothresholded using the “MinError(I)” method. Thresholded anti-ChAT and OK6-GAL4 > GFP images were subtracted to remove cell bodies from the OK6-GAL4 > GFP image, leaving only a mask that specified the dorsal region of the VNC neuropil (Supplementary Figures S1A–D). To generate ChAT and dVGAT ROIs, immunostaining was subjected to Laplacian transformation (smoothing scale = 4) using FeatureJ (Meijering, 1996–2024) and autothresholded using the “Default” method (Supplementary Figures S1E,I). ROIs were specified as occurring within the motor domain by subtracting the custom motor domain mask (Supplementary Figures S1E–L). The remaining ROIs (Supplementary Figures S1M,N) were then assessed using ImageJ’s in-house particle analysis function.

### 2.9 Single motor neuron morphological analysis

Larvae harboring transgenes for MultiColor FlpOut (Nern et al., 2015) with *Dscam2B*-GAL4 or *Exex*-GAL4 on *Dscam2* null and single isoform *Dscam2B* backgrounds were subjected to 5 min of heat shock at 37°C via water bath to stochastically label motor neurons. Individually labeled neurons were identified as being MN6/7-1b by following axons out of the VNC to their target muscles, the abdominal segment they projected to was documented to account for potential differences between segments. We successfully generated 11 controls, 8 *Dscam2^null^* and 32 *Dscam2B* singly labeled motor neurons. The sampling across neuromeres was roughly equivalent across genotypes except for *Dscam2^null^* (Supplementary Figure S3I). To analyze the projection ratio of MN6/7-1b motor neuron dendrites, z-stacks were max-projected in ImageJ and borders were traced around the perimeter of the rostral and caudal arbors of the basal dendrites. Rostral and caudal arbors were defined relative to the main neurite shaft originating from the cell body, from which dendritic projections arose. The max-projected areas of the rostral and caudal arbors were expressed as a percentage of the total basal dendritic tree area. Two motor neurons identified as MN6/7-1b in *Dscam2B* VNCs had to be discarded from analysis because their dendrites projected so aberrantly that we could not confidently categorize their arbors. It must be noted that any effects in the z axis cannot be identified by this type of analysis since we used an approach that relied on maximum projections. Furthermore, we did not analyze dendritic morphology, including length and branching.

### 2.10 Statistical analysis

A Shapiro–Wilk test was used to determine whether data assumed a normal distribution. To determine statistical significance between three or more groups that were all normally distributed, a one-way analysis of variance (ANOVA) was used, and multiple comparisons were corrected using Holm-Sidak’s *post hoc* test. To determine statistical significance between three or more groups where at least one group did not assume a normal distribution, a Kruskal-Wallis test with Dunn’s multiple comparisons test was used instead. Significance was determined at *p* < 0.05. All error bars represent mean ± 95% confidence interval. Lines in violin plots represent median (unbroken) and quartiles (dotted). Numbers (N) indicate number of larvae used or number of runs analyzed. Relevant details can be found in figure legends.

## 3 Results

### 3.1 Efficient larval locomotion requires regulated expression of *Dscam2* isoforms

**Figure 1:**
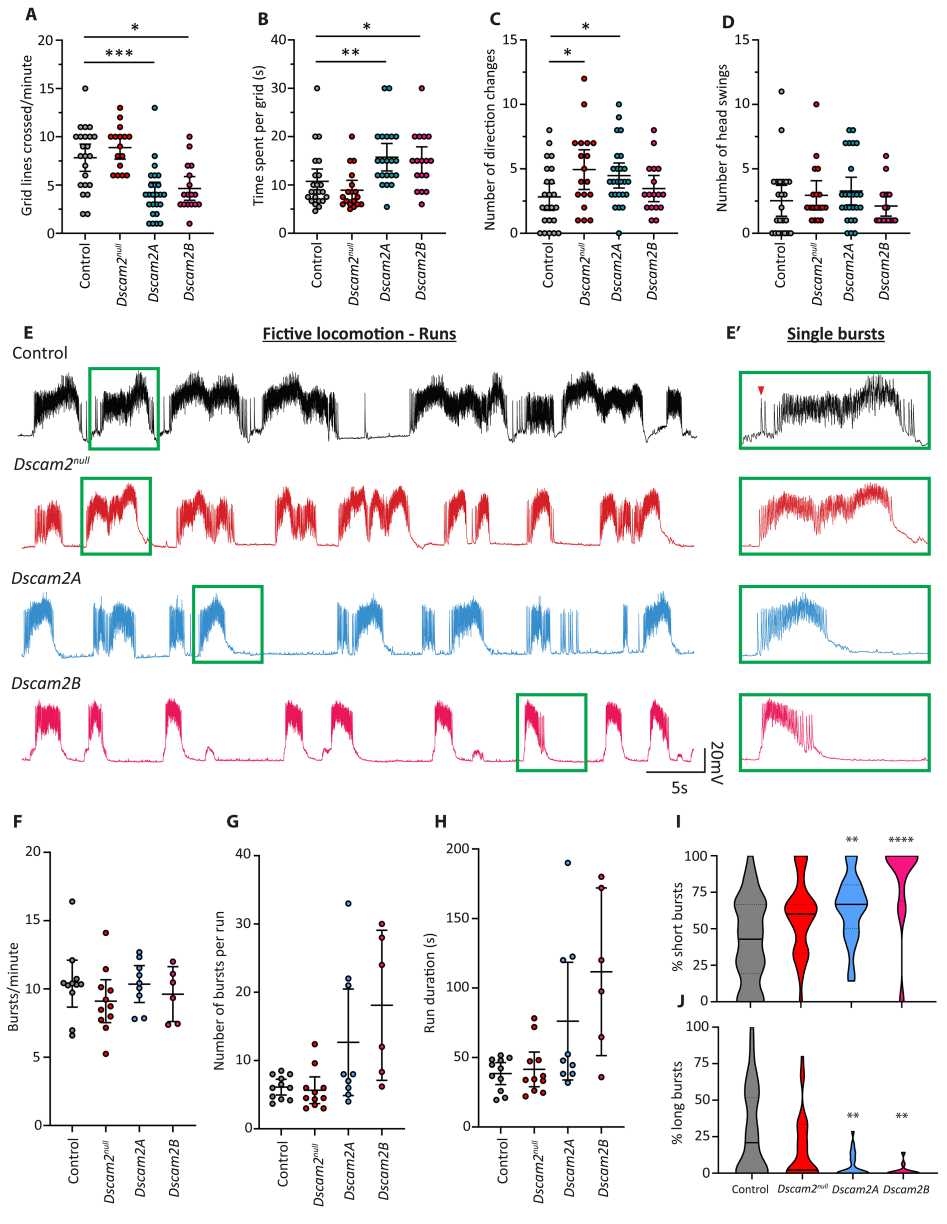
Locomotor and fictive locomotor defects following loss of *Dscam2* isoform diversity. **(A–D)** Motor behavior assessment of larvae lacking Dscam2 (*Dscam2^null^*) and larvae with ablated *Dscam2* alternative splicing, expressing only isoform A (*Dscam2A*) or only isoform B (*Dscam2B*). Quantification of the number of grid lines crossed by larvae in 60 s **(A)** and time spent within a single grid **(B)** revealed that *Dscam2A* and *Dscam2B* larvae had a locomotor defect relative to controls. Number of direction changes **(C)** was measured by counting the number of times larvae performed a head swing during forward linear locomotion and continued along the new path set by the head swing. Number of head swings **(D)** was measured by counting the number of times larvae performed a head swing while immobile. Groups analyzed using a Kruskal-Wallis test with Dunn’s multiple comparison, ^*^p < 0.05, ^**^p < 0.01, ^***^p < 0.001. *N* = number of larvae assessed; Control = 23, *Dscam2^null^* = 16, *Dscam2A* = 25, *Dscam2B* = 17. Error bars show 95% confidence interval. **(E,E′)** Representative traces of spontaneous compound EJPs recorded from larvae performing fictive locomotion. Bursts, which occur via sequential summing of EJPs, occur in a rhythmic fashion. Green insets show individual bursts within the runs **(E′)**. Red arrowhead shows a single EJP. Control = black, *Dscam2^null^* = red, *Dscam2A* = blue, *Dscam2B* = magenta. **(F–H)** Quantification of burst parameters within individual runs, where a run was defined by more than 3 consecutive bursts (within 10 s of each other). The frequency of bursts **(F)**, average number of bursts per run **(G)** and average duration of individual runs **(H)** is not statistically different between any of the measured genotypes. **(I,J)** Quantification of the composition of runs after separating bursts into short (<75 EJPs), medium (75–150 EJPs) and long (>150 EJPs) categories. *Dscam2A* and *Dscam2B* larvae displayed a statistically significant increase in the prevalence of short bursts **(I)** and a statistically significant decrease in the prevalence of long bursts **(J)**. Groups analyzed using a Kruskal-Wallis test with Dunn’s multiple comparison, ^**^p < 0.01, ^****^p < 0.0001. *N* = number of larvae assessed; Control = 11, *Dscam2^null^* = 11, *Dscam2A* = 9, *Dscam2B* = 6. Error bars show 95% confidence interval. Lines in violin plots represent median (unbroken line) and quartiles (broken lines).

Regulated alternative splicing of *Dscam2* is necessary for normal axon arborization (Lah et al., 2014), attaining appropriate numbers of synapses in the visual system (Kerwin et al., 2018) and synaptic physiology at the neuromuscular junction (Odierna et al., 2020). Whether eliminating isoform diversity impacts functional network output or behavior has not been explored. To address this, we chose to take advantage of the relative simplicity of *Drosophila* larvae, which exhibit highly stereotyped behavior. We investigated whether cell-specific expression of *Dscam2* isoforms is necessary for peristaltic locomotion using a grid assay, which involves counting the number of grid lines passed by larvae locomoting freely (Nichols et al., 2012). We tested animals lacking *Dscam2* (*Dscam2^null^*), and two separate knock-in lines that express a single isoform of *Dscam2* in all neurons that normally express this gene (*Dscam2A* and *Dscam2B*; Lah et al., 2014), thus eliminating cell-specific isoform expression. Larvae lacking *Dscam2* did not display any changes in the number of grid lines crossed compared to controls (Figure 1A). Conversely, both *Dscam2A* and *Dscam2B* larvae were sluggish and exhibited visible delays between peristaltic contractions. Quantification revealed that they crossed significantly fewer grid lines than controls (Figure 1A). They also spent much longer within individual grids (Figure 1B), highlighting their reduction in locomotor output. To further characterize motor behavior, we counted the number of times larvae performed a direction change during locomotion, characterized by execution of a head swing during linear forward locomotion and then continuing along the new path set by the head swing. We found that unlike the measures of gross locomotor output, *Dscam2^null^* larvae performed significantly more direction changes compared to controls (Figure 1C). Interestingly, we found that *Dscam2A* larvae also made more direction changes compared to controls, but that *Dscam2B* larvae did not (Figure 1C). The shared phenotype between *Dscam2^null^* and *Dscam2A* suggests that this phenotype could be caused by loss of *Dscam2B* in cells that normally express this isoform, as we have seen previously (Odierna et al., 2020). To test whether the direction changes in *Dscam2^null^* and *Dscam2A* larvae were caused by more baseline head swinging behavior we assessed the number of head swings performed while the larvae were immobile and not locomoting. We found that there was no difference in the number of head swings performed by larvae while stationary between all genotypes (Figure 1D). Taken together, these data indicate that although *Dscam2* itself is not required for locomotor output *per se*, regulated *Dscam2* isoform expression is necessary for efficient execution of locomotion. They also show that *Dscam2* may be important in regulating aspects of more complex larval behavior (such as that relating to decision making), since direction changes during locomotion likely reflect altered information processing in circuitry upstream of those directly governing locomotor output.

### 3.2 Motor neuron bursting is disrupted following loss of *Dscam2* isoform diversity

To gain additional insight into the locomotor defect of larvae expressing a single isoform of *Dscam2* (hereafter referred to as single isoform larvae), we turned to electrophysiological assessment of neuromuscular depolarizations *ex vivo*. This assay involves filleting larvae such that the central pattern generator (CPG) networks in the ventral nerve cord (VNC) remain intact and connected to the body-wall musculature (Kurdyak et al., 1994). During fictive locomotion, individual muscle fibers are impaled to measure compound EJPs at the neuromuscular junction (NMJ), generated by activated motor neurons. Most of the peripheral nerves must be severed to reduce mechanical disturbances, which precludes assessment of intersegmental coordination. This also means that the muscular contractions do not perfectly represent larval crawling. Importantly, despite removal of a large amount of sensory input to the VNC rhythmicity is still retained. Motor neuron firing leads to high frequency evoked EJPs at the NMJ called “bursts” (Kurdyak et al., 1994), which repeat at regular intervals to form what is called a “run” (Figures 1E,E′). Runs, and the bursts they are comprised of, arise from rhythmic CPG-dependent activation of motor neurons (Berni et al., 2012; Pulver et al., 2015) and as such provide a highly informative window into VNC circuit neurophysiology. We chose to focus our analysis on muscle 6, which is contacted by two motor neurons (1 s and 1b), of which one is *Dscam2B*-positive (MN6/7-1b). Though these motor neurons produce EJPs of different amplitude (Kurdyak et al., 1994), we could not confidently attribute individual EJPs to either one within each individual burst. This is because both motor neurons are co-activated during a burst and the EJPs sum over time. As such compound EJPs during a burst were not separated into different categories based on amplitude. The average number of bursts per minute (burst frequency) and the absolute number of consecutive bursts in a run were measured. Burst frequency, absolute number of bursts per run and burst duration were not different from controls in *Dscam2^null^*, *Dscam2A* or *Dscam2B* larvae (Figures 1F–H), indicating no gross disruptions to gross CPG rhythmicity following loss of *Dscam2* or its isoform diversity. The values we recorded for burst frequency closely matched those reported previously (Fox et al., 2006; McKiernan, 2013). Given that the speed of crawling is strongly determined by the frequency of peristaltic contractions, the lack of changes in burst frequency in the *Dscam2* single isoform larvae was unexpected. This suggested that there might be more subtle defects that disrupted their locomotor output.

We next investigated the properties of individual bursts within runs to explore in greater detail the influence of *Dscam2* isoform diversity on motor neuron activation. We initially assessed the average number of EJPs per burst, the average burst duration and the average frequency of EJPs per burst. None of the metrics displayed statistically significant differences between the genotypes (Supplementary Table S3). Intriguingly, the average number of EJPs per burst appeared consistently lower in both single isoform lines compared to control and *Dscam2^null^* larvae (Supplementary Table S3). Because of this, we hypothesized that there might be a more subtle change in the bursting of motor neurons in single isoform larvae, obscured by comparing averages across all bursts. We therefore separated bursts into short (<75 EJPs), medium (75–150 EJPs) and long (>150 EJPs) categories and calculated the composition of runs based on these categorizations. We found that both single isoform lines had runs that were composed of significantly more short bursts (Figure 1I) and fewer long bursts (Figure 1J). *Dscam2^null^* animals were no different to controls for all categories. Contraction of *Drosophila* larval muscles is not activated by action potentials but is instead directly driven by postsynaptic potentials (Peron et al., 2009). As such, the increase in short burst prevalence and reduction in long burst prevalence in single isoform larvae likely underlies their locomotor defect. Our data thus indicate that disrupted locomotion in single isoform animals arises from dysfunctional motor neuron activation.

### 3.3 Dscam2 protein is expressed in the larval VNC neuropil

**Figure 2:**
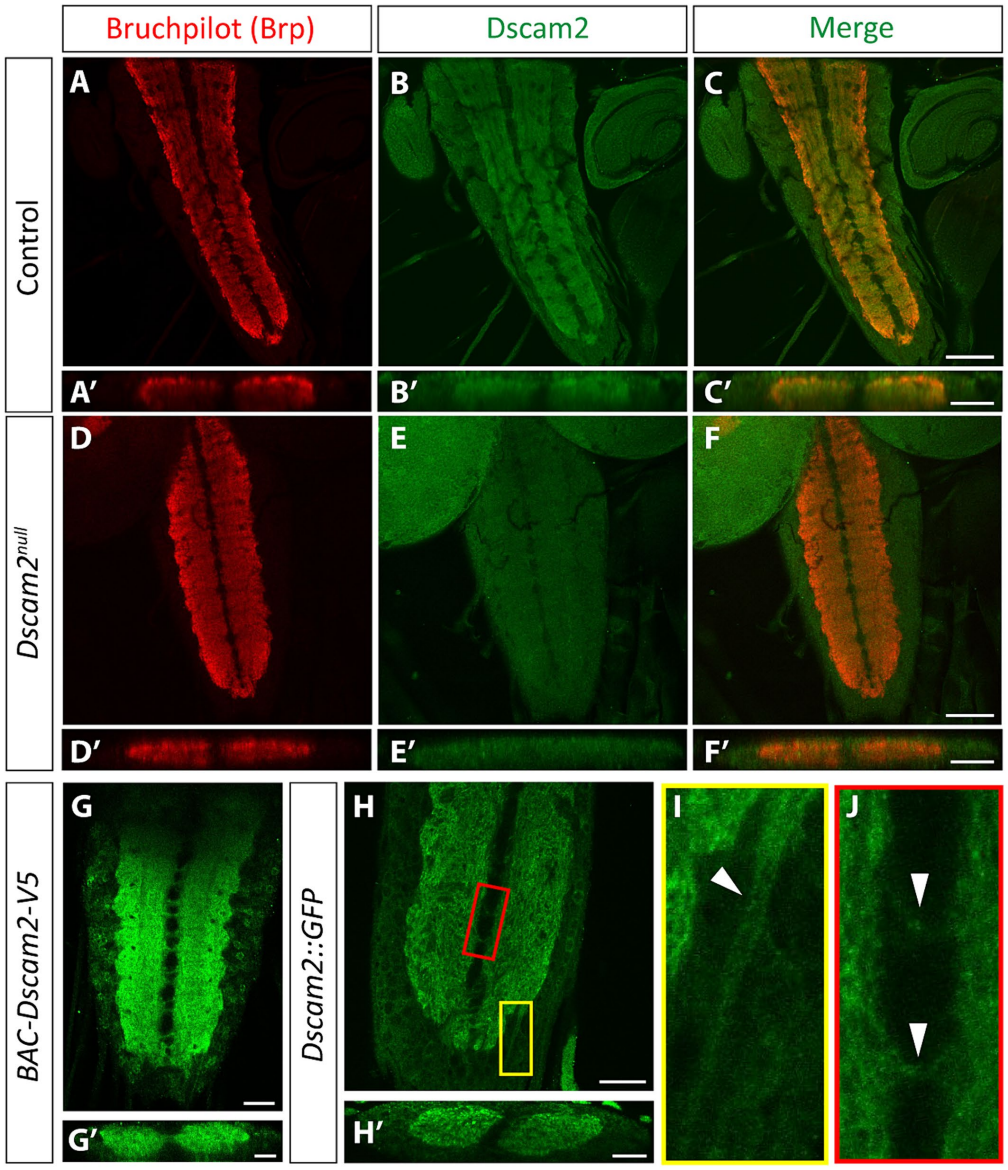
Dscam2 protein is expressed in the ventral nerve cord. **(A–F)** Representative images of immunostaining against Bruchpilot (Brp) and Dscam2 in the 3rd instar larval ventral nerve cord (VNC). Brp immunoreactivity (red) identifies the VNC neuropil (**A**, max-projected z-stack; **A′**, z-stack transverse reslice). Dscam2 immunoreactivity (green) using an antibody directed against the cytoplasmic region reveals protein localization to the VNC neuropil (**B,C**, max-projected z-stack; **B′,C′**, z-stack transverse reslice). Loss of signal in larvae lacking *Dscam2* (*Dscam2^null^*) confirms the specificity of the Dscam2 antibody **(D–F′)**. Scale bars in panels **(A–F)** are 50 μm. Scale bars in panels **(A′–F′)** are 20 μm. **(G,G′)** V5 immunoreactivity (green) in larvae harboring a V5-tagged Dscam2 (*BAC-Dscam2-V5*). A single optical slice is shown in panel **(G)** and a single optical transverse reslice is shown in panel **(G′)**. Scale bar in panel **(G)** is 20 μm. Scale bar in panel **(G′)** is 10 μm. **(H–J)** GFP fluorescence in the VNC of larvae expressing Dscam2::GFP-FLAG from the endogenous *Dscam2* locus (CRISPR knock-in). A single optical slice is shown in panel **(H)** and a single optical transverse reslice is shown in panel **(H′)**. Color-coded insets highlight GFP in presumed axon bundles containing sensory afferent and motor efferent axons (**I**, white arrowheads in yellow inset) and in commissural fascicles that contain axonal and dendritic neurites (**J**, white arrowheads in red inset). Scale bar in panel **(H)** is 20 μm. Scale bar in panel **(H′)** is 10 μm.

We reasoned that the altered motor neuron bursting in the single isoform lines could be explained by inappropriate Dscam2 interactions between VNC cells that normally express different isoforms. To visualize Dscam2 protein in the VNC, we used three separate methods. The first was immunolabeling using an antibody specific to the cytoplasmic region of Dscam2 (Millard et al., 2007). The second method was using a transgenic bacterial artificial chromosome (BAC) insertion containing a modified version of Dscam2 with a V5 tag in the extracellular region (see methods for details). The last method was using a CRISPR-generated endogenous GFP tag of Dscam2 in the intracellular region (see methods for details). We found that Dscam2 protein is expressed throughout the VNC neuropil, as outlined by Bruchpilot immunoreactivity (Figures 2A–C,A′–C′,G,H). Antibody specificity was validated in *Dscam2^null^* larvae, which showed no anti-Dscam2 immunoreactivity (Figures 2D–F,D′–F′). Expression of Dscam2 appeared evenly distributed throughout the VNC with no specific preference toward ventral or dorsal regions (Figures 2B′,G′,H′), which include sensory and motor domains, respectively (Landgraf et al., 2003). All three methods identified comparable regions in the VNC (Figures 2B,G,H), arguing against extensive localization differences between the extracellular and cytoplasmic domains. Using the GFP tag line we were able to identify signals in axon bundles (Figure 2I) and commissures (Figure 2J), which indicate neuritic localization of Dscam2 in VNC neurons. Thus, Dscam2 protein is present in the larval VNC in regions where interneurons and motor neurons are expected to interact with each other.

### 3.4 Different *Dscam2* isoforms are expressed in premotor interneurons and motor neurons

**Figure 3:**
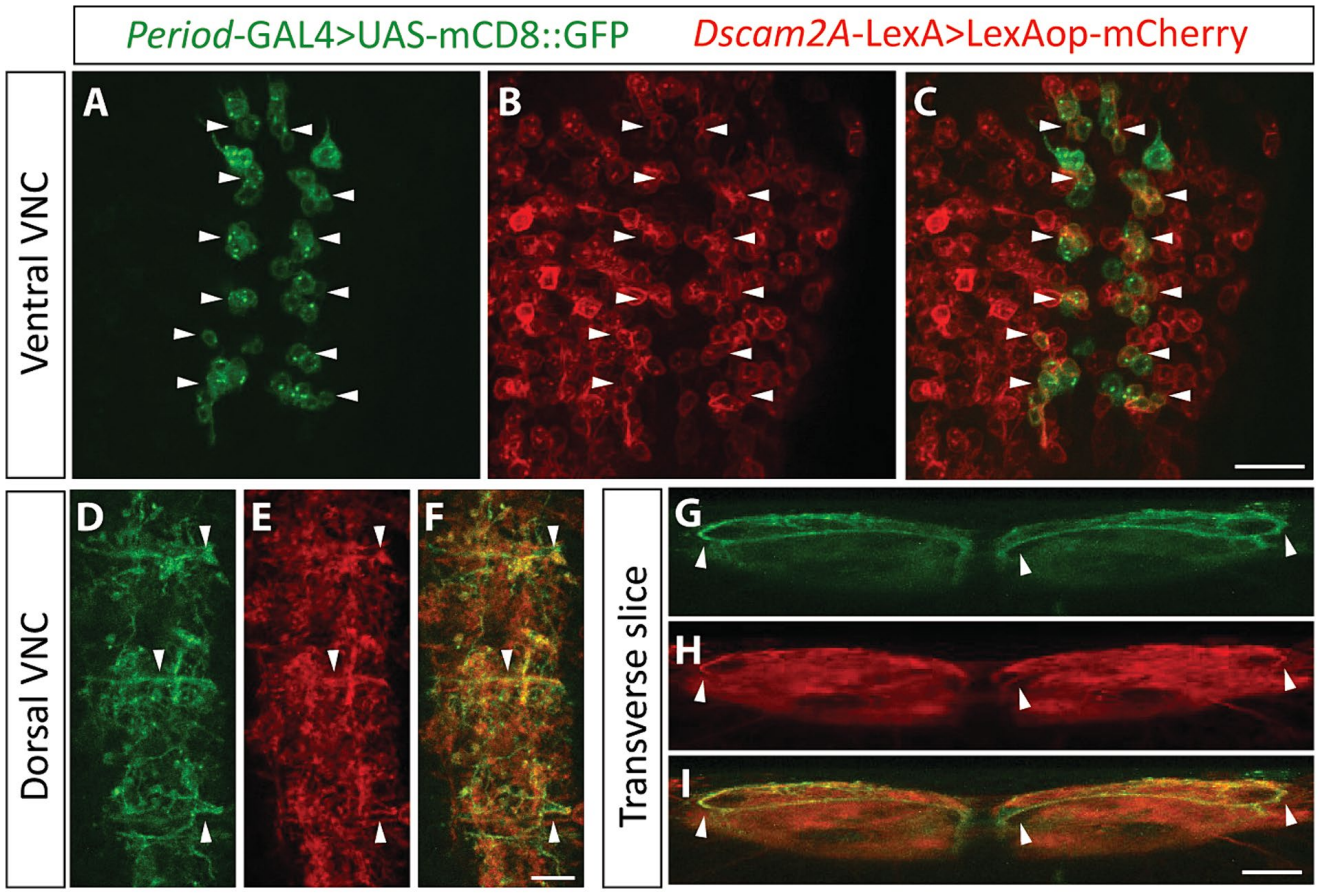
Looper premotor interneurons express *Dscam2A*. **(A–C)** Single optical slice from the ventral cortex of the nerve cord in larvae expressing *Period*-directed GFP (**A**, *Period*-GAL4 > UAS-mCD8::GFP) and *Dscam2A*-directed mCherry (**B**, *Dscam2A*-LexA>LexAop-mCherry). Merged image in panel **(C)** shows that multiple *Period*-positive interneurons express *Dscam2A* (white arrowheads). Note that the ventral cortex of the nerve cord is where the cell bodies of period-positive median segmental interneurons (PMSIs, otherwise known as “loopers”) reside. Scale bar is 20 μm. **(D–F)** Expression of *Period*-directed GFP **(D)** and *Dscam2A*-directed mCherry **(E)** in the dorsal region of the VNC neuropil, where looper interneurons form synaptic contacts with motor neurons. Merged image in panel **(F)** shows colocalization in many neurites arborizing throughout the motor domain. Scale bar is 10 μm. **(G–I)** Transverse optical slice of the VNC showing the characteristic ventro-dorsal axonal projections of looper interneurons in *Period*-directed GFP (**G**, white arrowheads) are also identifiable using *Dscam2A*-directed mCherry (**H**, white arrowheads). Merged image in panel **(I)** shows close overlap between *Period*-directed GFP and *Dscam2A*-directed mCherry in presumed looper axons (white arrowheads). Scale bar is 10 μm.

Having found expression of Dscam2 protein in the VNC, including the motor domain, we recognized the possibility that removal of *Dscam2* isoform diversity might promote unwanted interactions between premotor interneurons and motor neurons. For this to be the case, premotor interneurons would need to express *Dscam2A*. To explore this possibility, we performed co-labeling using previously described reporters to identify *Dscam2A*-positive premotor interneurons. Of particular interest to us was the A02 interneuron population, also known as *Period*-positive median segmental interneurons (PMSIs) or “loopers” (Kohsaka et al., 2014) for the following reasons: (1) GFP Reconstitution Across Synaptic Partners (GRASP) experiments have revealed that looper axon terminals are in close membrane contact with motor neuron dendrites (Kohsaka et al., 2014), (2) Optogenetic activation of loopers directly generates glutamate-mediated inhibitory postsynaptic currents in MN6/7-1b motor neurons (MacNamee et al., 2016), and, (3) Transmission electron microscopy reconstructions have confirmed that A02g and A02e loopers form synaptic connections with MN6/7-1b motor neurons (Zarin et al., 2019). We used the GAL4 system to mark *Period*-positive neurons and the LexA system to mark *Dscam2A*-positive neurons and found that 72–76% of *Period*-positive VNC interneurons express *Dscam2A* (Figures 3A–C). We were able to confidently identify loopers within the population of *Dscam2A*-expressing cells based on their unique ventro-dorsal projections (Figures 3D–I) confirming that they express *Dscam2A*. We also investigated the expression of *Dscam2A* in a population of VNC interneurons called Glutamatergic Ventro-Lateral Interneurons (GVLIs). Although GVLIs are second-order premotor interneurons, their axons project directly into the VNC motor domain and are in close enough proximity with motor neuron dendrites to induce GRASP activation (Itakura et al., 2015). We used R26F05-LexA to label GVLIs and used the GAL4 system to label *Dscam2A*-positive neurons. Using this strategy, we found that 100% of GVLIs express *Dscam2A* (Supplementary Figures S2A–F). Thus, first- and second-order premotor interneurons in close membrane proximity with *Dscam2B*-positive motor neurons express *Dscam2A*. This includes looper premotor interneurons, which are direct synaptic partners of MN6/7-1b motor neurons.

### 3.5 Inappropriate Dscam2 interactions produce motor neuron dendritic targeting defects

**Figure 4:**
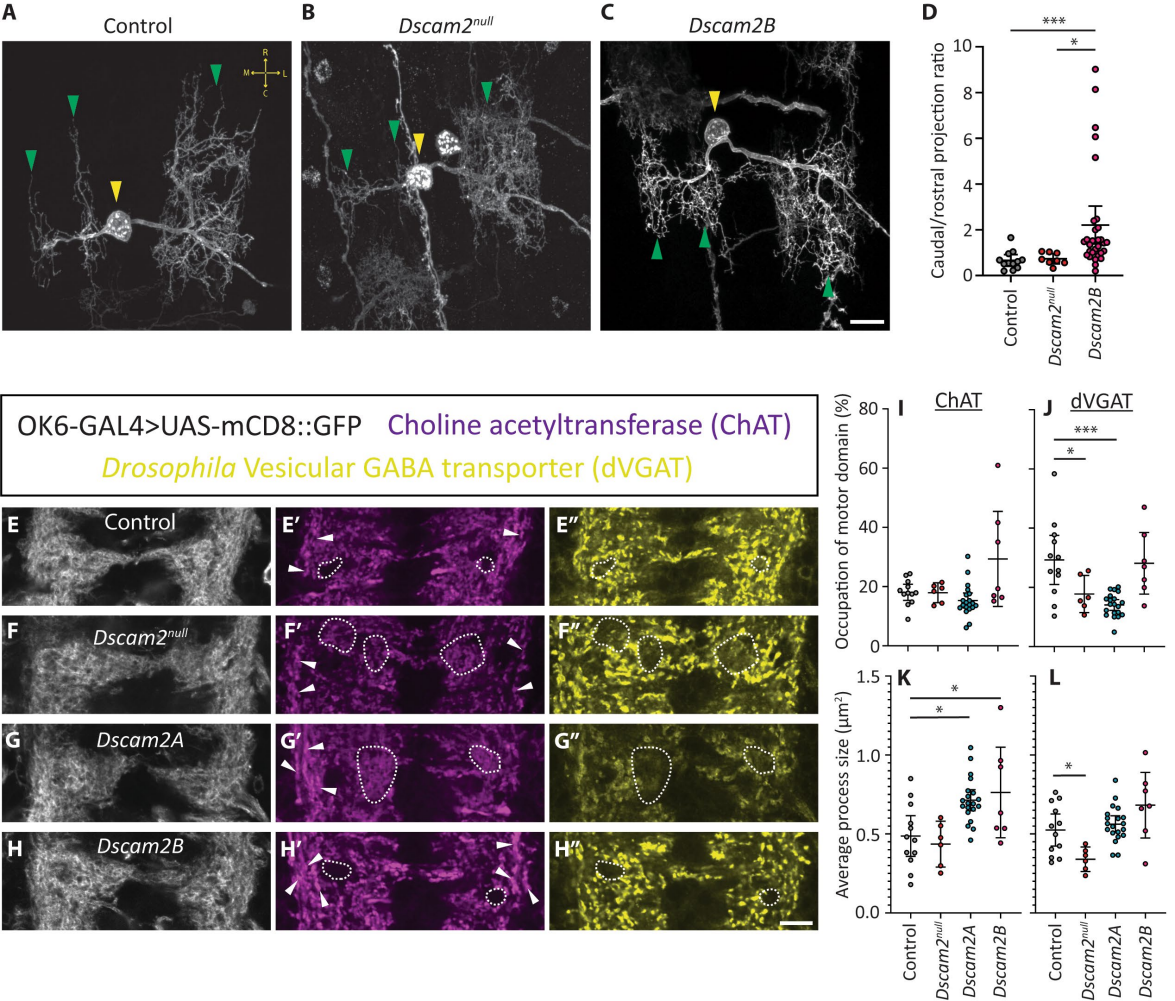
Inappropriate Dscam2 interactions disrupt motor neuron dendrite patterning and inputs to the motor domain. **(A–C)** Representative example of singly labeled MN6/7-1b neurons in control, *Dscam2^null^* and *Dscam2B* larvae using a MultiColor FlpOut strategy. Yellow arrowheads indicate singly labeled MN6/7-1b motor neurons. Green arrowheads indicate the three distinct dendritic arrays formed by MN6/7-1b. Yellow arrows in panel **(A)** show the general orientation of MN6/7-1b in the ventral nerve cord; R = Rostral, C = Caudal, M = medial, L = lateral. Scale bar is 10 μm. **(D)** Quantification of max-projected z-stacks by outlining basal dendritic arbors revealed that the ratio of dorsal-caudal projections was significantly higher in the *Dscam2B* MN6/7-1b motor neurons compared to control and *Dscam2^null^*. Groups analyzed using a Kruskal-Wallis test with Dunn’s multiple comparison, ^*^p < 0.05, ^***^p < 0.001. *N* = number of motor neurons analyzed (1 motor neuron per larva); Control = 11, *Dscam2^null^* = 8, *Dscam2B* = 32. Error bars show 95% confidence interval. **(E–H″)** Representative images of triple labeled ventral nerve cords with OK6-GAL4 > UAS-mCD8::GFP fluorescence, anti-Choline acetyltransferase (ChAT) immunoreactivity and anti-*Drosophila* Vesicular GABA transporter (dVGAT) immunoreactivity. Dotted outlines show regions with reduced dVGAT processes. Note that in controls and single isoform B larvae these regions are often devoid of ChAT processes as well and overlap with regions of reduced OK6-GAL4 > GFP fluorescence, suggesting they are structural gaps in the neuropil. In *Dscam2^null^* and single isoform A larvae, regions of poor dVGAT innervation are often innervated with ChAT processes, showing they are part of the neuropil experiencing reduced GABAergic innervation. White arrowheads show longitudinal ChAT+ve projections that are normally punctate but appear specifically enlarged on the lateral and medial edges of the motor domain in single isoform A and B larvae. Scale bar is 10 μm. **(I–L)** Quantification of thresholded ChAT and dVGAT processes in the motor domain. The percentage of the motor domain occupied by ChAT processes was not different between all genotypes **(I)** but *Dscam2^null^* and *Dscam2A* had significantly less motor domain dVGAT processes relative to controls **(J)**. The average ChAT+ve process size was significantly higher in *Dscam2A* and *Dscam2B* relative to controls **(K)** and the average size for dVGAT processes was significantly lower in *Dscam2^null^* relative to controls **(L)**. Groups analyzed using a one-way ANOVA with Holm-Sidak’s multiple comparisons test, ^*^p < 0.05, ^***^p < 0.01. *N* = number of larvae assessed; Control = 12, *Dscam2^null^* = 6, *Dscam2A* = 20, *Dscam2B* = 7. Error bars show 95% confidence interval.

Inappropriate interactions between premotor interneurons and MN6/7-1b in the single isoform lines could result in a range of phenotypes that might explain the altered motor neuron bursting phenotype. We reasoned that a change in motor neuron dendrite structure would serve as the simplest explanation for a change in their output. We therefore set out to assess the dendritic arbors of MN6/7-1b motor neurons. We used a FlpOut strategy to identify single MN6/7-1b motor neurons in control, *Dscam2^null^* and *Dscam2B* backgrounds. Single motor neurons from *Dscam2A* larvae were not generated since motor neurons do not express Dscam2A and most of the phenotypes assessed thus far were similar with both isoforms. We successfully generated 11, 8 and 32 singly labeled MN6/7-1b neurons in controls, *Dscam2^null^* and *Dscam2B*, respectively. Control MN6/7-1b dendrites formed 3 distinct dendritic arrays that arborized asymmetrically within the VNC, with a preference to project in the rostral direction (Figure 4A; Supplementary Figures S3A–D). MN6/7-1b neurons in *Dscam2^null^* larvae appeared largely similar to controls with respect to the gross projection of their dendritic arbors (Figure 4B), consistent with our previous finding that loss of *Dscam2* does not impact locomotor output. This was not true for *Dscam2B* MN6/7-1b neurons, which sometimes projected their arbors in an opposite direction to controls (Figure 4C; Supplementary Figures S3E,F). We quantified the percentage area of basal dendritic arbor for max-projected z-stacks of MN6/7-1b neurons and found that in controls, 75% of the arbor projected rostrally to the main shaft on average and 25% projected caudally. In *Dscam2^null^* this was not different, but in *Dscam2B* approximately one third of the assessed neurons projected predominantly in the caudal direction (Figure 4C; Supplementary Figures S3E–H). Quantification of the average dendritic projection ratios of MN6/7-1b motor neurons revealed that *Dscam2B* was significantly different to controls and *Dscam2^null^* (Figure 4D). The dendritic phenotype of two *Dscam2B* motor neurons was so severe that they had to be omitted from the analysis; their arbors projected aberrantly outside of the segment where the main shaft arose, which may represent a less prevalent but consequential phenotype in terms of locomotion. To account for potential segmental effects on morphology, we segregated motor neurons based on segment. Although we did not have enough singly labeled motor neurons per segment to perform a statistical analysis, segregating the data this way allowed us to visualize whether the effect was segment specific. This visualization revealed that the effect appeared strongest in motor neurons residing in rostral neuromeres (Supplementary Figure S3J). This possibly implies a spatial specificity for the inappropriate Dscam2 interactions, though additional work is needed to confirm this due to our low sampling frequency relative to each neuromere. Overall, these results demonstrate that inappropriate Dscam2-mediated interactions produce dendritic targeting defects in motor neurons, which may cause them to receive incorrect inputs.

### 3.6 Loss of *Dscam2* isoform diversity disrupts excitatory and inhibitory projections into the motor domain

Having found dendritic targeting defects in motor neurons, we wondered whether there were also broader disruptions to connectivity in the VNC motor domain. Specifically, we were curious about how inappropriate Dscam2 interactions might tip the organizational balance of excitation and inhibition. Although we found *Dscam2A* expression in glutamatergic interneurons (loopers and GVLIs), which are inhibitory in the larval VNC, it is difficult to assess glutamatergic input to the motor domain because motor neurons are also glutamatergic. This makes it impossible to guarantee that glutamatergic processes in the motor domain are exclusively from interneurons. To circumvent this issue, we instead chose to assess cholinergic and GABAergic projections to the motor domain, which are excitatory and inhibitory, respectively. Although we have not confirmed *Dscam2* expression in cholinergic or GABAergic interneurons, we expect that many interneurons other than loopers or GVLIs express *Dscam2A* based on our previous observations (Odierna et al., 2020). We measured the projections of excitatory and inhibitory interneurons to motor neurons using antibodies that recognize choline acetyltransferase (ChAT) and the *Drosophila* vesicular GABA transporter (dVGAT). Imaging revealed discrete cholinergic and GABAergic processes (Figures 4E′,E″). Because we were specifically interested in inputs to motor neurons, we used OK6-GAL4-based GFP fluorescence to direct our analysis. OK6-GAL4 labels most motor neurons and is not expressed in sensory neurons, which allows specific labeling of the motor domain of the VNC (Sanyal, 2009). ChAT and dVGAT immunoreactivity were thresholded to generate ROIs representing cholinergic and GABAergic projections, respectively (Supplementary Figures S1E–L). The percentage of the OK6-GAL4-based VNC neuropil mask occupied by ChAT processes was unchanged across all genotypes (Figures 4E–H,E′–H′,I). Despite this, there was a significant increase in the average size of ChAT processes in *Dscam2A* and *Dscam2B* animals relative to controls (Figure 4K). This increased area seemed to arise predominantly from ChAT+ve longitudinal projections on the lateral and medial edges of the motor domain, which otherwise appeared punctate in control and *Dscam2^null^* animals (Figures 4G′,H′). Assessment of dVGAT processes revealed that GABAergic innervation of the motor domain was significantly decreased in *Dscam2^null^* and *Dscam2A* larvae (Figures 4E–H,E″–H″,J). This reduction was often observed as regions within the neuropil with no dVGAT projections or very sparse innervation with small processes, particularly in *Dscam2^null^* larvae (Figures 4F″,G″). In line with this observation, the size of dVGAT processes in *Dscam2^null^* larvae was significantly smaller than controls (Figure 4L). Together, these data demonstrate that proper projections from excitatory and inhibitory interneurons to the motor domain rely on *Dscam2*.

## 4 Discussion

Dscam2 regulates multiple processes crucial to neuronal development (Millard et al., 2010; Lah et al., 2014; Kerwin et al., 2018; Odierna et al., 2020). It performs these functions by promoting cell–cell adhesion or repulsion via isoform-specific homophilic interactions (Millard et al., 2007; Tadros et al., 2016). Here, we asked whether cell-specific regulation of *Dscam2* isoform expression is required for the functional output of the neuromotor system. Our behavioral assessment of *Drosophila* larvae revealed that loss of *Dscam2* itself does not grossly impact locomotor output. However, removal of cell-specific *Dscam2* isoform expression produces a strong disruption to locomotion. The phenotypic similarity between both single isoform lines demonstrates that there is no specific role for either isoform in modulating CPG-controlled locomotion. Rather, Dscam2A and Dscam2B likely regulate VNC circuit formation and refinement in specific populations of neurons; when isoform diversity is removed, interactions between these populations produce disruptions. Electrophysiological assessment of synaptic depolarizations during *ex vivo* fictive locomotion demonstrated that both *Dscam2A* and *Dscsam2B* motor neurons produced fewer EJPs upon activation than controls. *Drosophila* larval body-wall muscles do not fire action potentials but instead produce graded contractions based on calcium influxes induced directly by EJPs (Peron et al., 2009). The weaker motor neuron bursts in single isoform larvae therefore likely explains their disrupted locomotion (due to weaker activation of muscle). This result provides evidence that the contributions of regulated *Dscam2* isoform expression to synaptic connections (Lah et al., 2014; Kerwin et al., 2018) and synaptic physiology (Odierna et al., 2020) have functional consequences on network output and refinement of motor behavioral programs. This finding mirrors work on the vertebrate *DSCAM*, which is becoming increasingly recognized as a key player in the organization of motor system circuitry (Ma et al., 2020; Lemieux et al., 2021).

Assessment of single motor neurons revealed that eliminating *Dscam2* isoform diversity results in changes to the dendritic arborization patterns of motor neuron dendrites, which may cause them to receive incorrect inputs from neurons in neighboring neuromeres. Further, both single isoform A and B larvae displayed a notable increase in the size of cholinergic projections to the motor domain. These defects likely arise from inappropriate interactions between motor neurons and premotor interneurons that project into the motor domain. In this study, we identify that GVLI and looper interneurons express *Dscam2A*. Given that loopers are directly presynaptic to MN6/7-Ib motor neurons (which express *Dscam2B*), they serve as a good candidate for the source of the inappropriate Dscam2-mediated interactions. Alternate expression of Dscam2 isoforms in these neurons likely permits them to use Dscam2 to refine their innervation patterns without inducing unwanted interactions between each other. Thus, our results demonstrate how alternative splicing of *Dscam2* can be employed to ensure efficient neuromotor patterning.

We found that a subset of phenotypes was shared by *Dscam2^null^* and *Dscam2A* larvae. The number of direction changes during locomotion was higher in both lines but not in *Dscam2B* larvae. GABAergic input to the motor domain was also reduced in *Dscam2^null^* and *Dscam2A* larvae. We have previously observed shared phenotypes between *Dscam2^null^* and *Dscam2A* larvae and have suggested that this occurs due to loss of Dscam2B in cells that normally express this isoform (Odierna et al., 2020). Our previous findings demonstrate that Dscam2B suppresses synaptic strength and that Dscam2A cannot replace this function. The observation of Dscam2A insufficiency in the VNC reinforces the idea that the two isoforms are not interchangeable and therefore further reinforces the importance of *Dscam2* alternative splicing in nervous system function. Although it remains unclear exactly how loss of Dscam2B impacts larval behavior it appears to be associated with decreased GABAergic projections into the motor domain, possibly indicating altered inhibitory signaling from upstream circuitry. Expression of *Dscam2* in embryos begins in most VNC cells right before hatching (Odierna et al., 2020), which is in line with a critical period for activity-dependent network refinement in embryos (Giachello and Baines, 2015; Ackerman et al., 2021). This particular developmental period is also marked by the emergence of multi-step behavioral patterns such as those required to self-right (Crisp et al., 2008). As such, *Dscam2* may be involved in fine-tuning networks that underlie complex behaviors or decision making. More work will be needed, however, to clarify how it does this.

## Data availability statement

The raw data supporting the conclusions of this article will be made available by the authors, without undue reservation.

## Ethics statement

The manuscript presents research on animals that do not require ethical approval for their study.

## Author contributions

GO: Conceptualization, Data curation, Formal analysis, Investigation, Writing – original draft, Writing – review & editing. SK: Investigation, Writing – review & editing. GS: Investigation, Writing – review & editing. SM: Conceptualization, Funding acquisition, Project administration, Resources, Supervision, Writing – review & editing.

## Footnotes

^1^ https://bacpacresources.org/library.php?id=445
