# Supplementary material for: Drosophila larval motor patterning relies on regulated alternative splicing of Dscam2
Source: Front Mol Neurosci. 2024 Jul 18;17:1415207. doi: 10.3389/fnmol.2024.1415207 (PMC11292952; doi:10.3389/fnmol.2024.1415207)
Supplement: Supplementary file 1 [file Table1.DOCX]

Supplementary Material

# Supplementary Figures and Tables

## Supplementary Table 1

Table S1. Fly lines used in this study.

| **Line** | **Description** | **Source** |
| --- | --- | --- |
| Dscam2^null-1^ | *Dscam2* knock out | Millard et al. (2007) |
| Dscam2^10A-A^ | *Dscam2* single isoform A | Lah et al. (2014) |
| Dscam2^10A-D^ | *Dscam2* single isoform A | Lah et al. (2014) |
| Dscam2^10B-A^ | *Dscam2* single isoform B | Lah et al. (2014) |
| Dscam2^10B-D^ | *Dscam2* single isoform B | Lah et al. (2014) |
| Dscam2A-GAL4 | *Dscam2A* splice trap – GAL4 | Lah et al. (2014) |
| Dscam2B-GAL4 | *Dscam2B* splice trap – GAL4 | Lah et al. (2014) |
| Dscam2A-LexA | *Dscam2A* splice trap – LexA | Tadros et al. (2016) |
| Dscam2B-LexA | *Dscam2B* splice trap – LexA | Tadros et al. (2016) |
| Dscam2^GFP-FLAG^ | GFP-tagged Dscam2 – intracellular domain | This study |
| BAC-Dscam2-V5 | V5-tagged Dscam2 – extracellular domain | This study |
| OK6-GAL4 | *RapGAP1*-based GAL4 | Aberle et al. (2002) |
| Exex-GAL4 | *Exex-*based GAL4 | Broihier and Skeath (2002) |
| Period-GAL4 | *Period*-based GAL4 | Plautz et al. (1997) |
| R26F05A-LexA | Split LexA expressed in GVLIs | Jenett et al. (2012) |
| 10xUAS-FRT>STOP>FRT-myr::smGFP-V5 | V5 spaghetti monster GFP for MultiColor FlpOut | Nern et al. (2015) |
| 10xUAS-FRT>STOP>FRT-myr::smGFP-myc | myc spaghetti monster GFP for MultiColor FlpOut | Nern et al. (2015) |
| hsFlp | Heat shock flippase | Nern et al. (2015) |
| UAS-mCD8::GFP | mCD8::GFP under control of a UAS |  |
| UAS-mCD8::RFP | mCD8::RFP under control of a UAS |  |
| LexAop-mCherry | mCherry under control of a LexAop |  |

## Supplementary Table 2

Table S2. Antibodies used in this study.

| **Antibody** | **Dilution** | **Source** |
| --- | --- | --- |
| Cy3-conjugated goat anti-HRP | 1:500 | Jackson Immunoresearch Laboratories |
| Cy5-conjugated goat anti-HRP | 1:500 | Jackson Immunoresearch Laboratories |
| nc82 mouse anti-Brp | 1:100 | Developmental Studies Hybridoma Bank |
| CHATB1 mouse anti-ChAT | 1:500 | Developmental Studies Hybridoma Bank |
| Rabbit anti-dVGAT | 1:500 | Gift from David Krantz - Fei et al. (2010) |
| Mouse anti-GFP | 1:500 | Bio-Rad |
| Rabbit anti-Dscam2 | 1:1000 | Millard et al. (2007) |
| V5-tag:DyLight anti-mouse 550 | 1:500 | AbD Serotec |
| Alexa Fluor 488-conjugated goat anti-mouse IgG | 1:800 | Life Technologies |
| Alexa Fluor 647-conjugated goat anti-mouse IgG | 1:800 | Life Technologies |
| Cy3-conjugated donkey anti-rabbit IgG | 1:800 | Life Technologies |

## Supplementary Table 3

Table S3. Additional parameters from electrophysiological assessment of fictive locomotion.

|  | **Control**  N=11 | ***Dscam2^null^***  N=11 | ***Dscam2A***  N=9 | ***Dscam2B***  N=6 |
| --- | --- | --- | --- | --- |
| **Number of EJPs per burst** | 112.4$\pm$41.6 | 111.9$\pm$13.7 | 79.8$\pm$30.8 | 68.84$\pm$21.7 |
| **Burst duration (s)** | 3.2$\pm$1 | 3.6$\pm$1.2 | 2.9$\pm$0.9 | 2.2$\pm$0.8 |
| **EJP frequency per burst (Hz)** | 34.3$\pm$8.9 | 28.8$\pm$4.6 | 27.9$\pm$6.1 | 33.9$\pm$10.5 |

## Supplementary Figure 1

##
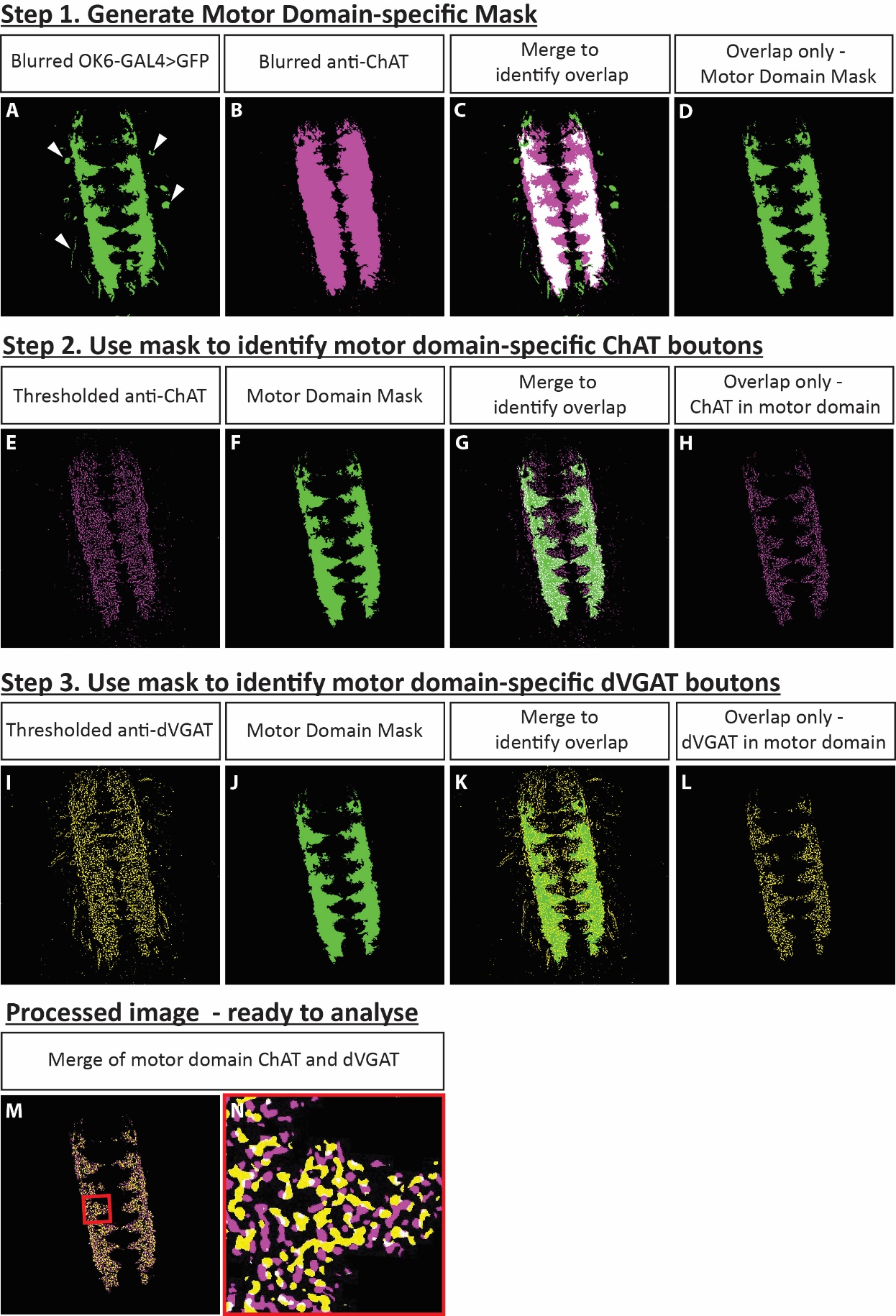


**Figure S1.** **Specification of ChAT and dVGAT bouton analysis to the motor domain.** (**A-D**) Generation of the custom motor domain mask, which specifies the dorsal region of the ventral nerve cord (VNC) neuropil. OK6-GAL4-based GFP fluorescence (OK6-GAL4>GFP) labels motor neuron cell bodies, dendrites and axons. Cell bodies and axons, shown with white arrowheads in panel (**A**), introduce nonspecific regions of interest (ROIs) so need to be removed to generate a pure motor domain mask. To achieve this, anti-Choline acetyltransferase (ChAT) immunoreactivity is blurred substantially to serve as a VNC neuropil mask (**B**). ChAT is ideal for this function given it is strongly expressed in the neuropil and rarely overlaps with OK6-GAL4>GFP in the cortex or in axons since axonal expression of ChAT from sensory neurons enters the VNC via ventral pathways. Overlap between **A** and **B** is shown in **C**, which is used to generate the final mask (**D**). (**E**-**N**) Cholinergic and GABAergic boutons in the VNC are generated by subjecting anti-ChAT and anti-*Drosophila* Vesicular GABA transporter (dVGAT) immunoreactivity to Laplacian transformation and thresholding (**E** and **I**, respectively). Boutons in the motor domain are specified by findings those that overlap with the motor domain mask (**F**, **G**, **J**, **K**) for each slice in the z-stack of whole VNCs. This leaves only ChAT and dVGAT boutons in the motor domain (**H** and **L**, respectively). Merge of the resultant images shows that processes retain largely non-overlapping patterns, as expected (**M**, **N**).

## Supplementary Figure 2


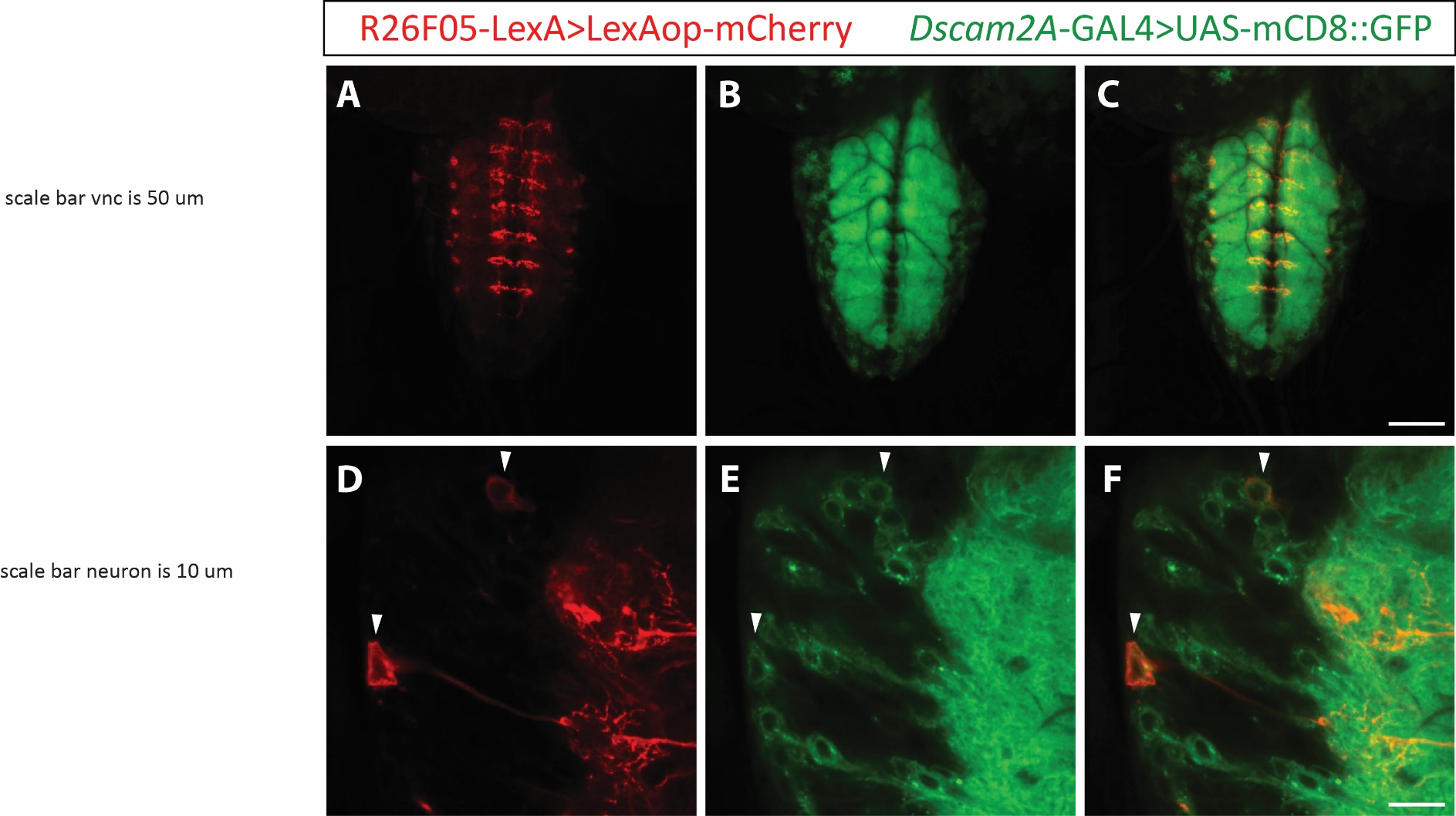


**Figure S2.** **Glutamatergic Ventro-Lateral Interneurons express *Dscam2A*.** (**A-C**) Max-projected z-stack of the ventral nerve cord (VNC) in larvae expressing R26F05-directed mCherry (**A**, R26F05-LexA>LexAop-mCherry) and *Dscam2A*-directed GFP (**B**, *Dscam2A*-GAL4>UAS-mCD8::GFP). Merged image in **C** shows substantial overlap between labels. Scale bar is 50 µm. (**D**-**F**) High power single optical slice of RF26F05-LexA>mCherry and *Dscam2A*-GAL4>GFP in the ventro-lateral region of the VNC cortex, where the cell bodies of Glutamatergic Ventro-Lateral Interneurons (GVLIs) reside. Cell body and projections of single GVLIs (**D**, white arrow heads) and *Dscam2A*+ve interneurons (**E**) are visible in the ventro-lateral region of the VNC. Merged image in **F** shows clear expression of *Dscam2A* in GVLIs, as indicated by white arrowheads. Scale bar is 10 µm.

## Supplementary Figure 3

**
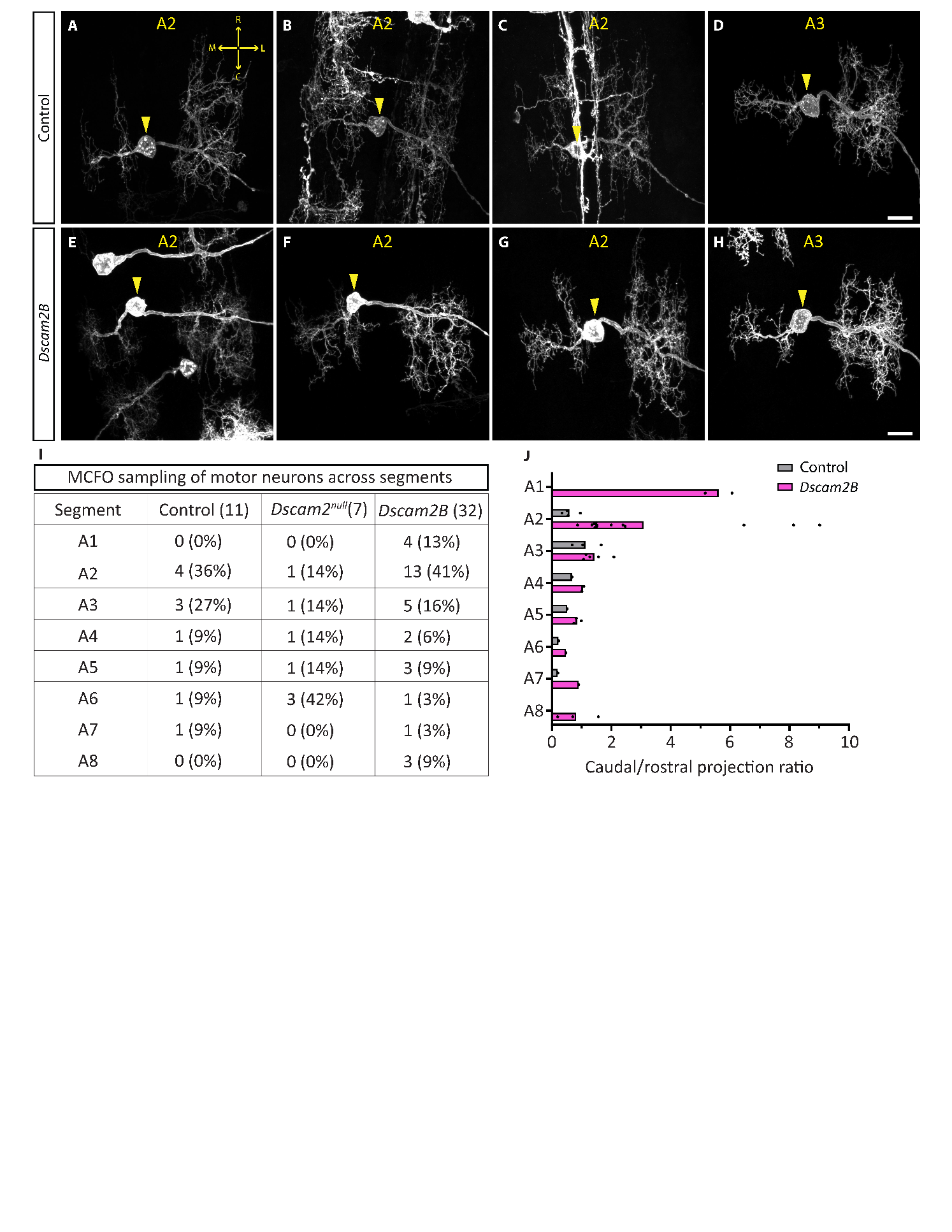
**

**Figure S3.** **Dendritic defects in *Dscam2B* larvae.** Additional examples of single MN6/7-1b motor neurons labelled using MultiColour FlpOut. (**A-D**) Control MN6/7-1b motor neurons tended to project their dendrites in a rostral direction with very little variability. (**E-H**) *Dscam2B* single isoform MN6/7-1b motor neurons displayed a high degree of variability with respect to their dendritic projections. Some projected predominantly in the caudal direction (**E**, **F)** whereas others appeared similar to controls but with somewhat exaggerated caudal projections (**G, H**). Abdominal segment is indicated using yellow text in each image. Yellow arrowheads indicate singly labelled MN6/7-1b motor neurons. Yellow arrows in (**A**) show general orientation of the motor neurons in ventral nerve cord; R = Rostral, C = Caudal, M = medial, L = lateral. Scale bar is 10 µm. (**I**) Breakdown of motor neuron sampling across abdominal segments using a stochastic MultiColor FlpOut (MCFO) labelling strategy. Segments were confirmed by following motor neurons axons out to the abdominal segment they innervated. (**J**) Visualization of motor neuron dendrite arbor caudal/rostral projection rations broken down by segment. Statistical tests were not performed due to low sample numbers at individual segments. However, this visualization does indicate a possible enhancement of the dendrite phenotype in more rostral segments like A1 and A2.

# References

Aberle, H., Haghighi, A. P., Fetter, R. D., McCabe, B. D., Magalhães, T. R., & Goodman, C. S. (2002). wishful thinking Encodes a BMP Type II Receptor that Regulates Synaptic Growth in Drosophila. *Neuron*, *33*(4), 545-558. <https://doi.org/https://doi.org/10.1016/S0896-6273(02)00589-5>

Broihier, H. T., & Skeath, J. B. (2002). Drosophila Homeodomain Protein dHb9 Directs Neuronal Fate via Crossrepressive and Cell-Nonautonomous Mechanisms. *Neuron*, *35*(1), 39-50. <https://doi.org/https://doi.org/10.1016/S0896-6273(02)00743-2>

Fei, H., Chow, D. M., Chen, A., Romero-Calderón, R., Ong, W. S., Ackerson, L. C., Maidment, N. T., Simpson, J. H., Frye, M. A., & Krantz, D. E. (2010). Mutation of the Drosophila vesicular GABA transporter disrupts visual figure detection. *Journal of Experimental Biology*, *213*(10), 1717-1730. <https://doi.org/10.1242/jeb.036053>

Jenett, A., Rubin, Gerald M., Ngo, T.-T. B., Shepherd, D., Murphy, C., Dionne, H., Pfeiffer, Barret D., Cavallaro, A., Hall, D., Jeter, J., Iyer, N., Fetter, D., Hausenfluck, Joanna H., Peng, H., Trautman, Eric T., Svirskas, Robert R., Myers, Eugene W., Iwinski, Zbigniew R., Aso, Y., . . . Zugates, Christopher T. (2012). A GAL4-Driver Line Resource for Drosophila Neurobiology. *Cell Reports*, *2*(4), 991-1001. <https://doi.org/https://doi.org/10.1016/j.celrep.2012.09.011>

Lah, G. J., Li, J. S., & Millard, S. S. (2014). Cell-specific alternative splicing of Drosophila Dscam2 is crucial for proper neuronal wiring. *Neuron*, *83*(6), 1376-1388. <https://doi.org/10.1016/j.neuron.2014.08.002>

Millard, S. S., Flanagan, J. J., Pappu, K. S., Wu, W., & Zipursky, S. L. (2007). Dscam2 mediates axonal tiling in the Drosophila visual system. *Nature*, *447*(7145), 720-724. <https://doi.org/10.1038/nature05855>

Nern, A., Pfeiffer, B. D., & Rubin, G. M. (2015). Optimized tools for multicolor stochastic labeling reveal diverse stereotyped cell arrangements in the fly visual system. *Proceedings of the National Academy of Sciences*, *112*(22), E2967-E2976. <https://doi.org/doi:10.1073/pnas.1506763112>

Plautz, J. D., Kaneko, M., Hall, J. C., & Kay, S. A. (1997). Independent Photoreceptive Circadian Clocks Throughout &lt;em&gt;Drosophila&lt;/em&gt. *Science*, *278*(5343), 1632. <https://doi.org/10.1126/science.278.5343.1632>

Tadros, W., Xu, S., Akin, O., Yi, Caroline H., Shin, Grace J.-e., Millard, S. S., & Zipursky, S. L. (2016). Dscam Proteins Direct Dendritic Targeting through Adhesion. *Neuron*, *89*(3), 480-493. <https://doi.org/https://doi.org/10.1016/j.neuron.2015.12.026>
